# Completion of the AAV Structural Atlas: Serotype Capsid Structures Reveals Clade-Specific Features

**DOI:** 10.3390/v13010101

**Published:** 2021-01-13

**Authors:** Mario Mietzsch, Ariana Jose, Paul Chipman, Nilakshee Bhattacharya, Nadia Daneshparvar, Robert McKenna, Mavis Agbandje-McKenna

**Affiliations:** 1 Department of Biochemistry and Molecular Biology, Center for Structural Biology, McKnight Brain Institute, College of Medicine, University of Florida, Gainesville, FL 32610, USA; mario.mietzsch@ufl.edu (M.M.); aej51@miami.edu (A.J.); pchipman@ufl.edu (P.C.); rmckenna@ufl.edu (R.M.); 2 Biological Science Imaging Resource, Department of Biological Sciences, Florida State University, Tallahassee, FL 32306, USA; nilakshee.bhattacharya@duke.edu (N.B.); nd15b@my.fsu.edu (N.D.)

**Keywords:** AAV, serotype, capsid, cryo-EM, genome packaging, gene delivery

## Abstract

The capsid structures of most Adeno-associated virus (AAV) serotypes, already assigned to an antigenic clade, have been previously determined. This study reports the remaining capsid structures of AAV7, AAV11, AAV12, and AAV13 determined by cryo-electron microscopy and three-dimensional image reconstruction to 2.96, 2.86, 2.54, and 2.76 Å resolution, respectively. These structures complete the structural atlas of the AAV serotype capsids. AAV7 represents the first clade D capsid structure; AAV11 and AAV12 are of a currently unassigned clade that would include AAV4; and AAV13 represents the first AAV2-AAV3 hybrid clade C capsid structure. These newly determined capsid structures all exhibit the AAV capsid features including 5-fold channels, 3-fold protrusions, 2-fold depressions, and a nucleotide binding pocket with an ordered nucleotide in genome-containing capsids. However, these structures have viral proteins that display clade-specific loop conformations. This structural characterization completes our three-dimensional library of the current AAV serotypes to provide an atlas of surface loop configurations compatible with capsid assembly and amenable for future vector engineering efforts. Derived vectors could improve gene delivery success with respect to specific tissue targeting, transduction efficiency, antigenicity or receptor retargeting.

## 1. Introduction 

Adeno-associated viruses (AAV) are single-stranded DNA packaging viruses of the *Parvoviridae* and belong to the genus *Dependoparvovirus* [1]. Vectors based on AAVs are being developed and used as gene delivery biologics to treat a large variety of monogenetic diseases [2]. Thirteen human and primate AAV serotypes, and numerous genomic isolates have been described and have been assigned to six clades A–F or individual clonal isolates [3]. The virions of the AAVs are composed of non-enveloped capsids with T = 1 icosahedral symmetry and diameters of ≈260 Å [4]. They are assembled from 60 viral proteins (VPs): VP1 (≈82 kDa), VP2 (≈73 kDa), and VP3 (≈61 kDa) in an approximate 1:1:10 ratio [5]. The VPs share a common C-terminus that includes the entirety of VP3. Compared to VP3, VP1 and VP2 are extended at their N-termini with a shared ≈65 amino acid (aa) region and additional ≈137 aa N-terminal to VP2 in the case of VP1 (VP1u). The N-terminal regions of VP1 and VP2 contain conserved elements required for AAV infectivity such as a phospholipase A2 (PLA2) domain, a calcium-binding domain, and nuclear localization signals [6,7]. Overall, the VP1 amino acid sequence identity of the AAV serotypes varies between 57 and 99% [8].

The capsid structures of several natural human and primate AAV serotypes, AAV1-AAV6, AAV8, AAV9, AAVhu.37, AAVrh.8, AAVrh.10, and AAVrh.39 have been determined by either X-ray crystallography and/or cryo-electron microscopy (cryo-EM) [9,10,11,12,13,14,15,16,17,18,19]. Regardless of the method of structure determination, only VP3 of the AAVs, except for the first ≈15 aa, are structurally ordered. The VP3 structure consists of an anti-parallel, eight-stranded (βB to βI) β-barrel motif, with the BIDG sheet forming the inner surface of the capsid. An additional strand, βA, runs anti-parallel to the βB strand. Furthermore, all AAVs conserve an α-helix (αA) located between βC and βD. Between the individual β-strands, large loops are inserted that are characterized by high sequence and structure variability among the AAVs. These loops form the exterior surface of the capsid and are named after their flanking β-strands. For example, the HI loop is flanked by the βH and βI strands. The sequence variability of different AAVs results in alternative conformations of these loops, which result in AAV serotype-specific capsid surface features. Nine regions of significant diversity at the apex of these loops have been defined as variable regions (VRs) by structural alignments [15]. Despite the structural differences of the VRs, the overall capsid morphology is conserved. These include cylindrical channels at the icosahedral 5-fold symmetry axes, formed by the DE-loops (VR-II), surrounded by a depression largely outlined by the HI-loops. The 5-fold channel is believed to be the route of genomic DNA packaging and VP1u externalization during endo/lysosomal trafficking following cell entry [20,21]. At the 2-fold symmetry axes, depressions are flanked by protrusions surrounding the 3-fold symmetry axes, and raised capsid regions between the 2- and 5-fold axes are termed 2/5-fold walls. The 3-fold region as well as the 2/5-fold wall have been identified as receptor binding sites for many AAV serotypes and serve as determinants of cell and tissue tropism. Among the cellular receptors are sialic acids [22,23,24], heparan sulfate proteoglycans (HSPG) [25,26,27,28,29], terminal galactose [30,31], sulfated N-acetyl-lactosamine [32], AAVR [33], laminin [34], αvβ1 integrin [35], αvβ5 integrin [36], the hepatocyte growth factor receptor [37], the fibroblast growth factor receptor [38], and platelet-derived growth factor receptor [39]. In addition to receptor binding, the surface of the capsid, including the 5-fold region, displays antigenic sites for antibodies raised by the host immune response [40].

In this study, the structures of the AAV7, AAV11, AAV12, and AAV13 capsids were determined by cryo-EM in an effort to complete the panel of available structures for the defined AAV serotypes. The empty and genome-containing capsid structures of these four AAV serotypes were reconstructed to be between 2.54 to 3.15 Å resolution. All density maps displayed well-defined amino acid side chain densities and showed the characteristic AAV capsid features, including the channels at the 5-fold axes, depressions at the 2-fold and surrounding the 5-fold axes, and protrusions that surround the 3-fold axes. The comparison of the empty (no DNA) and full (genome packaged) capsid structures showed no structural differences of the VP monomer except for an ordered nucleotide at the previously described nucleotide (nt) binding pocket in the case of the full capsids and alternative side chain orientations [17]. Compared to AAV2, significant structural differences were observed primarily at the 3-fold protrusions and the 2/5-fold wall due to aa insertions or deletions as well as sequence differences. This characterization of the structures of AAV7, AAV11, AAV12, and AAV13, completes the library for the defined serotypes. This provides a means to functionally annotate their capsids and a visual platform to aid recombinant DNA vector engineering for improved gene delivery applications.

## 2. Materials and Methods 

### 2.1. AAV Production and Purification

The AAV7, AAV11, AAV12, and AAV13 producer plasmid *cap* genes were synthesized by GenArt (Thermo Fisher, Waltham, MA, USA) and subcloned into a plasmid with the AAV2 *rep* gene to generate pR2V7, pR2V11, pR2V12, and pR2V13, respectively. Recombinant AAV7, AAV11, AAV12, and AAV13 vectors, with a packaged luciferase gene, were produced by the triple transfection of HEK293 cells, utilizing pTR-UF3-Luciferase, pHelper (Stratagene, San Diego, CA, USA), and either pR2V7, pR2V11, pR2V12, or pR2V13, and harvested 72 h post transfection as previously described [41]. The cleared lysates containing AAV7, AAV12, and AAV13 capsids were purified by AVB Sepharose and AAV11 by POROS Capture Select AAVX affinity chromatography as previously described [42]. Sample purity and capsid integrity were monitored by SDS-PAGE and negative- stain electron microscopy using a Spirit microscope (FEI, Hillsboro, OR, USA).

### 2.2. Cryo-Electron Microscopy Data Collection and 3D Image Reconstruction

For each of the purified AAV capsids, 3.5 μL was applied to a glow-discharged Quantifoil copper grid with 2 nm continuous carbon support over holes (Quantifoil R 2/4 400 mesh), blotted, and vitrified using a Vitrobot Mark 4 (FEI, Hillsboro, OR, USA) at 95% humidity and 4 °C. The capsid distribution and ice quality of the grids were screened in-house using an FEI Tecnai G2 F20-TWIN microscope (FEI) operated under low-dose conditions (200 kV, ≈20e^−^/Å^2^). Images were collected on a GatanUltraScan 4000 CCD camera (Gatan, Pleasanton, CA, USA). Grids deemed suitable for high-resolution data collection were used for collecting micrograph movie frames using the Leginon application [43] on a Titan Krios electron microscope. The microscope was operated at 300 kV and data were collected on a Gatan K3 direct electron detector. During data collection, a total dose of ≈60 e^−^/Å^2^ was utilized for 45 to 71 movie frames per micrograph (Table 1). The movie frames were aligned using MotionCor2 with dose weighting [44]. All datasets were collected as part of the NIH “Southeastern Center for Microscopy of MacroMolecular Machines (SECM4)” project. For the three-dimensional image reconstruction, the cisTEM software package was utilized [45] and the data were processed as described previously [46]. The sharpened density maps were inspected using Coot and Chimera [47,48]. The −90 Å^2^/0 Å^2^ sharpened maps were utilized for assignment of the amino acid main- and side chains. The resolution of the cryo-reconstructed density maps for empty (no DNA) and genome-containing AAV7, AAV11, AAV12, and AAV13 capsids were estimated based on a Fourier Shell Correlation of 0.143 (Table 1).

### 2.3. Model Building and Structure Refinement

Three-dimensional (3D) homology models of AAV7, AAV11, AAV12, and AAV13 VP3 were generated with the protein structure homology-modeling server Swiss model (https://swissmodel.expasy.org) [49] using their amino acid sequences (NCBI accession numbers YP_077178, AAT46339, ABI16639 and ABZ10812, respectively) and supplying the VP3 structures of AAV8 (PDB accession number: 2QA0) for AAV7, AAV4 (2G8G) for AAV11 and AAV12, and AAV3 (3KIC) for AAV13 as templates [9,14,15]. A T = 1 60-mer capsid coordinate model was generated from the respective VP3 with the VIPERdb2 Oligomer generator subroutine by icosahedral matrix multiplication [50]. The 60-mer capsid models of each AAV were docked into their cryo-reconstructed density maps by rigid body rotations and translations using the ‘fit in map’ subroutine within UCSF-Chimera [48]. This application uses a correlation coefficient (CC) calculation to assess the quality of the fit between the map generated from the model and the reconstructed map. During the model fitting, the voxel (pixel) size of each reconstructed map was adjusted to optimize the CC between the models and maps. The fitted models were exported relative to the respective map for further use. Each map was resized to the voxel size determined in Chimera using the “e2proc3D.py” subroutine in EMAN2 [51] and then converted to the CCP4 format using the program MAPMAN [52]. A VP monomer was extracted from each 60-mer and the side- and main chains were adjusted into the maps by manual building and the real-space refinement subroutine in Coot [47]. The adjusted capsid model was refined against the map utilizing the rigid body, real space, and B-factor refinement subroutines in Phenix [53]. Capsid model refinement was alternated with visualization and adjustment of VP side- and main chains using Coot while maintaining model geometry as well as rotamer and Ramachandran constraints [47]. The CC and refinement statistics, including root mean square deviations (RMSD) from ideal bond lengths and angles (Table 1), were analyzed using Phenix [53]. 

### 2.4. AAV Capsid Structure Comparison 

The Cα positions of the ordered amino acids within the VP3 atomic coordinates for each of the AAVs were superposed using secondary structure matching (SSM) in Coot [54]. This SSM subroutine also generates a list of the Cα–Cα distances between the aligned structures, which was used to calculate the overall root mean square deviation (RMSD). Deviations between non-overlapping Cα positions, because of residue deletions or insertions, were measured using the distance tool in Coot. Structural identity was determined using PDBeFold (https://www.ebi.ac.uk/msd-srv/ssm/) and calculated as the number of aligned residues (<1.0 Å apart) divided by the total number of residues. Amino acid sequence alignments of the different AAV serotypes were done utilizing the sequence alignment option in VectorNTI (Invitrogen, Carlsbad, CA, USA).

### 2.5. Structure Accession Numbers 

The full and empty AAV7, AAV11, AAV12, and AAV13 cryo-EM reconstructed density maps and models built for their capsids were deposited in the Electron Microscopy Data Bank (EMDB) with accession numbers EMD-23190/PDB ID 7L5U (AAV7 full), EMD-23189/PDB ID 7L5Q (AAV7 empty), EMD-23202/PDB ID 7L6E (AAV11 full), EMD-23203/PDB ID 7L6F (AAV11 empty), EMD-23200/PDB ID 7L6A (AAV12 full), EMD-23201/PDB ID 7L6B (AAV12 empty), EMD-23204/PDB ID 7L6H (AAV13 full), EMD-23205/PDB ID 7L6I (AAV13 empty), respectively.

## 3. Results and Discussion

### 3.1. The Structures of AAV7, AAV11, AAV12, and AAV13 Capsids Completes the Serotype List

**Figure 1:**
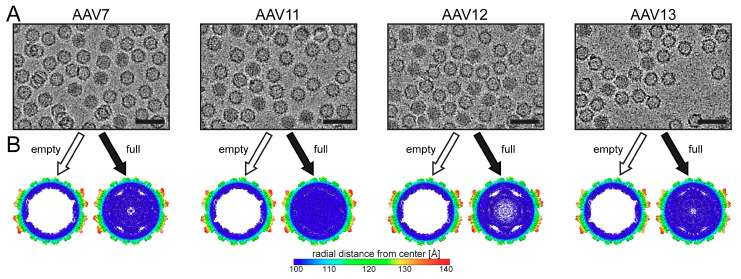
Cryo-electron microscopy (cryo-EM) reconstruction of genome containing (full) and empty AAV7 and AAV11–AAV13 capsids. (**A**) Cryo-electron micrographs showing the presence of full capsids (dark appearance) and empty (light appearance). Scale bar: 50 nm. (**B**) Cross-sectional views of the reconstructed maps determined by cryo-EM reconstruction from full and empty capsids contoured at a sigma (σ) threshold level of 0.9. The reconstructed maps are radially colored (blue to red) according to radial distance to the particle center. This figure was generated using UCSF-Chimera [48].

The capsid structures of AAV serotypes 1–6 and 8–9 have been previously reported [9,10,11,12,13,14,15], leaving those of AAV7 and AAV10–13 yet to be determined. AAV10, a member of clade E [3], possesses just a single amino acid (aa) difference (A589T) within VP3 compared to AAVrh.39, for which the capsid structure has been determined [17]. Thus, the AAV10 capsid structure is likely identical to AAVrh.39, especially since the AAVrh.10 capsid, which has several aa differences, is already shown to be structurally identical to AAVrh.39 [17]. In contrast, AAV7 (has an 82 aa difference in VP3 vs. AAV8), AAV11 (109 aa vs. AAV4), AAV12 (109 aa vs. AAV4), and AAV13 (28 aa vs. AAV3) are substantially different to their closest sequence-related AAV serotype, as shown in the parentheses. Thus, to determine their capsid structures recombinant AAV7, AAV11, AAV12, and AAV13 vectors were produced by triple transfection of HEK293 cells followed by purification with AVB affinity chromatography in the case of AAV7, AAV12, and AAV13, and AAVX affinity chromatography in the case of AAV11, as described in the methods. While the affinity purification resulted in highly pure AAV capsid preparations, it did not separate empty (no genome) and genome-containing (full) capsids, and thus, both types of capsids were observed in cryo-EM micrographs (Figure 1A).

The distribution of the capsids in the micrographs enabled the independent structural determination of both empty and full capsids using 2D classification, as described previously [46], for each serotype. For AAV7, AAV11, AAV12, and AAV13, the empty/full structures were determined from 40,988/4695, 118,351/10,429, 220,137/40,764, and 56,962/6794 capsids, respectively, to 2.96/3.16, 2.86/3.15, 2.54/2.67, and 2.76/3.00 Å resolution (FSC 0.143), respectively (Table 1). For each of the AAV serotypes, the resolution of the full structures is slightly lower compared to the empty, which is most likely due to fewer capsids used in the reconstructions of the former. Direct comparison of the reconstructed empty and full maps for each AAV serotype in a cross-sectional view clearly showed the electron-dense filled interior of the genome-containing capsids, which is absent from the empty capsids (Figure 1B). Similar to previous observations of full AAV capsid density maps, the majority of the capsid interior is filled except for the region directly underneath the 5-fold channel [17,46]. It has been suggested that the dynamic and flexible VP1/VP2 common region and VP1u could be located in the area under the 5-fold channel in readiness to be externalized through the 5-fold channel, which is a structural rearrangement that is required for its PLA2 enzyme function during the viral life cycle [20].

### 3.2. The AAV7, AAV11, AAV12, and AAV13 Capsid Structures Conserved the AAV Features

**Figure 2:**
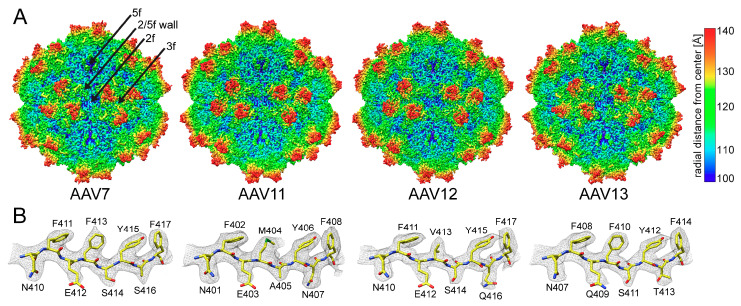
The AAV7 and AAV11–13 capsid structures. (**A**) The capsid surface density maps contoured at a sigma (σ) threshold level of 2.0. The maps are radially colored (blue to red) according to distance to the capsid center, as indicated by the scale bar on the right. The icosahedral 2-, 3-, and 5-fold axes as well as the 2/5-fold wall are indicated on the AAV7 capsid map. (**B**) The AAV7 and AAV11–13 amino acids modeled for the βG strand are shown inside their respective density maps (black mesh). The amino acid residues are as labeled and shown in stick representation and colored according to atom type: C = yellow, O = red, N = blue, S = green. This figure was generated using UCSF-Chimera [48].

Regardless of whether full or empty maps were analyzed, the different AAV serotypes displayed the characteristic morphological features of other AAVs, e.g., a channel at the 5-fold symmetry axes, trimeric protrusions that surround each 3-fold symmetry axis, and a depression at each 2-fold symmetry axis (Figure 2A). However, the exact morphology of the 3-fold protrusions varies between the different AAV serotypes, with much broader protrusions for AAV11 and AAV12 compared to AAV7 and AAV13. Similarly, the shape and orientation of the 2-fold depression of AAV11 and AAV12 differs from AAV7 and AAV13. 

The reconstructed maps of the four AAV serotypes, empty and full, showed well-ordered amino acid side-chain densities (Figure 2B) throughout the VP structure starting at aa position 218–220 (AAV7 numbering), which is comparable to the other currently determined AAV serotype capsid structures [9,10,11,12,13,14,15]. The only exception was the apex of surface loop VR-IV in AAV7, where aa 455–458 (GGTAG) were disordered, preventing the reliable placement of main- and side-chain residues. A similar disorder was previously observed in AAVrh.10 and AAVrh.39 that share the same or very similar sequence at the apex of the loop, GGTAG and GGTQG, respectively [17]. The glycines on both sides of the apex likely confer the flexibility of this loop and thus the cause of the lack of structural order. AAV11–13 do not possess this accumulation of glycines at this loop; thus, their loops were structurally ordered.

### 3.3. The Full Capsids of AAV7, AAV11, AAV12, and AAV13 Show Ordered Nucleotides

**Figure 3:**
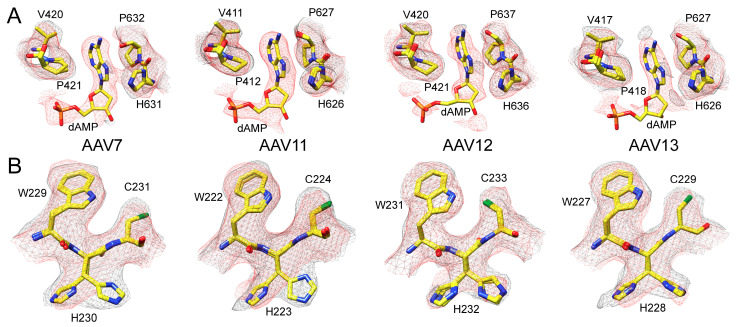
Empty and full Adeno-associated virus (AAV) density map differences. (**A**) The modeled AAV7 and AAV11–13 residues at the nucleotide binding pocket with their respective mesh density maps (black = empty, red = full). The extra density exclusively in the full maps was interpreted as an ordered nucleotide (deoxyadenosine monophosphate, dAMP). (**B**) Dual conformation of histidines (e.g., H230 in AAV7). This histidine adopts alternative side-chain conformations primarily in the absence of packaged DNA with the exception of AAV12. Atom colors: C = yellow, O = red, N = blue, S = green, P = orange. This figure was generated using UCSF-Chimera [48].

Similar to previous observations, a structural comparison of empty to the full capsids for the individual AAV serotypes showed them to be largely identical with overall Cα RMSDs ranging from ≈0.2 to 0.3 Å [17,46]. However, a major difference is the observation of weakly ordered density in the interior of the capsid maps in the full structures interpreted as the packaged genome (Figure 1B). This density extends into a pocket underneath the 3-fold symmetry axis and has been interpreted as deoxyadenosine monophosphate (dAMP), which is positioned between conserved prolines 421/632 and histidine 631 (AAV7 numbering) (Figure 3A). We hypothesized that the genome interacts with this 3-fold region of the interior capsid by binding within the pocket to two symmetry-related VP monomers [17]. Due to the imposed icosahedral symmetry during 3D image reconstruction and the fact that the genome cannot follow this symmetry, other nucleotides leading in and out of this pocket are weakly ordered and cannot be reliably modeled. We postulate that as reconstruction methods improve, relaxation of the enforced icosahedral symmetry in future structure determination efforts may allow the observation of a more ordered DNA structure. 

While the VP structures of empty and full capsids were largely identical, some alternative side-chain orientations, e.g., histidine 230 (AAV7 numbering), were observed. In the AAV serotype structures determined in this study, the histidine side chain preferred the “left” orientation in full capsids (Figure 3B). However, in AAV12 and AAV13, weak density was also observed toward the “right” orientation. In contrast, both orientations are equally adopted in empty capsids, except for AAV12, where the right orientation appears to be favored (Figure 3B). The dual conformation of H230 was previously observed in empty AAVrh.10 capsids [17]. While the cause of this difference between empty and full capsids is unknown, disordered density at low sigma level in the full maps, likely from the packaged genome, appears to contact the histidine side chain in the “right” orientation and thereby induce this preferred conformation of the side chain. Furthermore, H230 is located near the 5-fold symmetry axis, and the different conformation may be related to the observed differences underneath the 5-fold region in both types of capsids (Figure 1B).

### 3.4. The AAV7, AAV11, AAV12, and AAV13 Capsid Structures Display Diversity in Surface Loop Conformations

**Figure 4:**
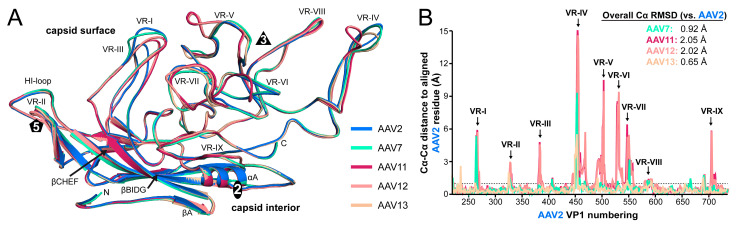
Structural comparison of AAV2, AAV7, and AAV11–13. (**A**) Structural superposition of AAV2 (blue), AAV7 (cyan), AAV11 (burgundy), AAV12 (salmon), and AAV13 (wheat) shown as ribbon diagrams. The positions of β-strands, the N- and C-terminus, the variable regions (VRs), and the icosahedral 2-, 3-, and 5-fold axis are indicated. This figure was generated using PyMol [60]. (**B**) Cα–Cα distance plot (in Å) for the AAV7 and AAV11–13 residues relative to AAV2 of the superposed viral protein (VP) structures. The VRs are indicated and the overall VP Cα-RMSD (root mean square deviation) compared to AAV2 shown. The dashed line marks the Cα–Cα distance variation of 1 Å.

The AAV7, AAV11–13 VP topologies conserve the core eight-stranded anti-parallel β-barrel (βB-βI), with the additional β-strand A and α-helix A (Figure 4A), as described previously for all other AAV structures [9,11,12,13,14,15,17,18,19,46,55,56,57,58,59]. When superposed, these core regions are homologous for the AAV serotypes (Figure 4A). Between the β-strands, the loops that form the surface of the capsids provide the structural variability among different AAVs. The nine VRs, I-IX previously defined [15], provide serotype-specific functions.

Compared to AAV2, the prototype serotype, some of the AAV7 and AAV11–13 surface loops showed only minor structural differences with Cα distances of ≤1 Å such as VR-VIII and the HI-loop (Figure 4B). AAV7 also shows minor structural differences compared to AAV2 in four additional loops (VR-III, VR-V, VR-VI, and VR-IX) and AAV13 in six additional loops (VR-I, VR-III, VR-V, VR-VI, VR-VII, and VR-IX), respectively. Structural variability (Cα distances of <3 Å) was also observed for the DE-loop/VR-II at the 5-fold symmetry axis for all analyzed AAV serotypes. The absence of major differences in the 5-fold region, which includes the HI-loop, are likely due to the common function these loops have to fulfill such as their role in DNA packaging and VP1u externalization [20,21]. 

Greater structural variability between AAV7 and AAV2 was seen in VR-I, VR-IV, and VR-VII due to single aa insertions (VR-I and VR-IV) or a single aa deletion (VR-VII) (Figure 4A,B). In AAV13, the only significant structural difference is observed in VR-IV, which is slightly shorter due to a single aa deletion compared to AAV2. In contrast, major structural variabilities (vs. AAV2) were seen in VR-I, VR-III, VR-IV, VR-V, VR-VI, VR-VII, and VR-IX for AAV11 and AAV12. Consequently, their overall Cα-RMSD for the entire VP is larger than that of AAV7 and AAV13 (Figure 4B). Most notably is the 5 aa insertion in VR-V for both AAV11 and AAV12, relative to AAV2, but also to AAV7 and AAV13 (Figure 4A). In addition, both AAV serotypes possess a single aa insertion in VR-IV and display a different conformation to the other AAV serotypes with the apex of the loop positioned over part of VR-V. This subloop of VR-V and the alternative conformation of VR-IV are responsible for the broader appearance of the 3-fold protrusions of the AAV11 and AAV12 capsids as described above (Figure 2A). On the side of the 3-fold protrusions VR-VI and VR-VII also showed significant differences with a 1 aa deletion in VR-VI and major structural variabilities in AAV11 and AAV12 (Figure 4A,B). Similarly, structural differences of VR-I, VR-III, and VR-IX lead to morphological differences at the 2/5-fold wall. VR-I takes a different conformation in AAV11 and AAV12 compared to AAV2 due to a 3 aa deletion. This is partially compensated by VR-III with a 2 aa insertion resulting in a broader loop without extending the height of the loop (Figure 4A). Finally, VR-IX of AAV11 and AAV12 displays a differential conformation without amino acid insertions or deletions relative to AAV2. This variation is located near the 2-fold symmetry axis, resulting in a slightly wider depression of the AAV11 and AAV12 capsid (Figure 2A). Their overall RMSD of the Cα coordinates for the entire VP of 2.05 and 2.02 Å (vs. AAV2) is greater than the overall Cα RMSD of AAV2 compared to AAV5, the most divergent AAV serotype, with a Cα RMSD of 1.8 Å [46].

### 3.5. The AAV7, AAV11, AAV12, and AAV13 Capsid Structures Display Clade-Specific Surface Features

**Figure 5:**
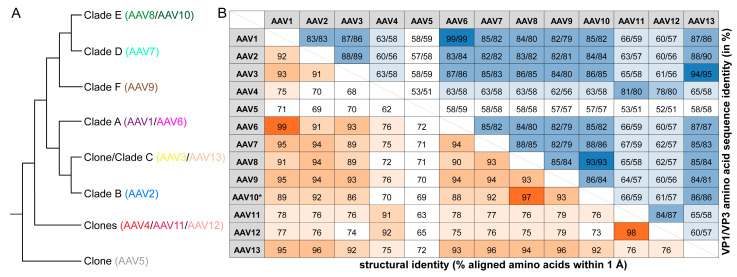
Relationship of the AAV serotypes. (**A**) Cladogram showing the assignment of the AAV serotypes to their clades as proposed by Gao et al. [3]. (**B**) Amino acid sequence identity of the AAV serotypes given as percentage for VP1 and VP3 or the structural identity as a percentage of aligned amino acids within 1Å when superposed. High values are colored in dark blue or orange and lower values in lighter shades of each color, respectively. * For AAV10, the VP structure of AAVrh.39 was utilized, which varies by a single aa from AAV10.

The clades for the AAV serotypes were proposed in 2004 [3] based on more than 100 unique isolates from human and non-human primates that were grouped based on their VP phylogenetical similarity (Figure 5A). AAV serotypes 10–13 were described after this study [28,61,62], and thus, they were originally not grouped into the clades. 

**Figure 6:**
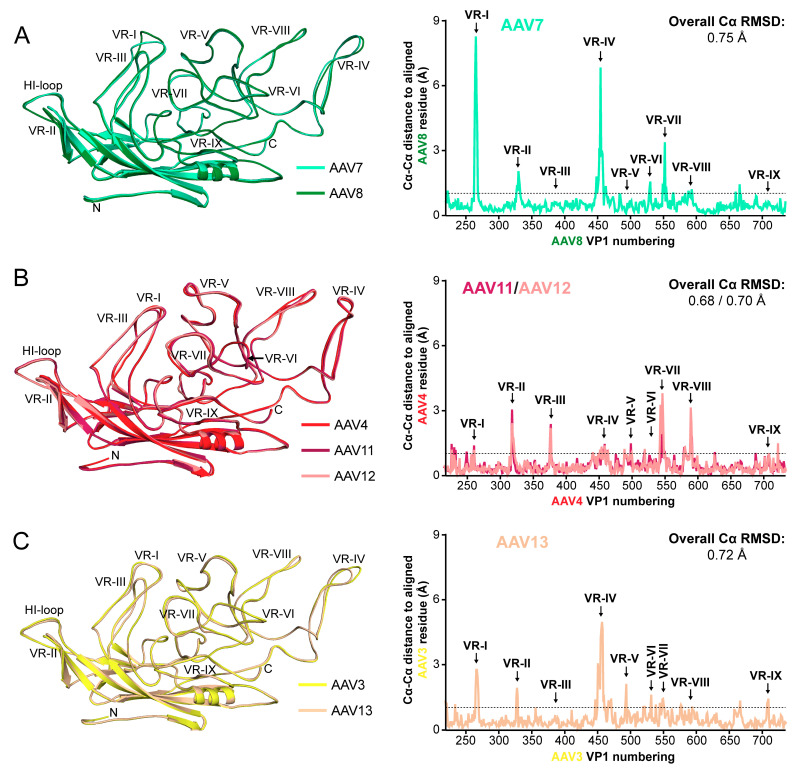
Structural comparison of AAV7 and AAV11–13 to their closest clade member. (**A**) Left—Structural superposition of AAV7 (cyan) and AAV8 (green) shown as ribbon diagrams. The positions of the N- and C-terminus and the variable regions (VRs) are indicated. This figure was generated using PyMol [60]. Right—Cα–Cα distance plot (in Å) for the AAV7 residues relative to AAV8 when the VP structures are superposed. The VRs are indicated, and the overall AAV7 VP Cα–RMSD compared to AAV8 is shown. The dashed line marks a Cα–Cα distance of 1 Å. (**B**) Structural comparison as in (A) for AAV4 (red), AAV11 (burgundy), and AAV12 (salmon). (**C**) Structural comparison as in (A) for AAV3 (yellow) and AAV13 (wheat).

AAV7 belongs to clade D (Figure 5A) and is closest related to the clade E members AAV8 and AAV10 based on VP1 or VP3 amino acid sequence ranging from 85 to 88% aa sequence identity (Figure 5B). When AAV7 is superposed onto AAV8, the overall Cα RMSD is 0.75 Å with a structural identity of 93% (Figure 5B and Figure 6A), which is slightly lower than the comparison to AAV2 described above at 0.92 Å. However, the 94% structural identity of AAV7 compared with AAV2 is slightly higher than for AAV7 vs. AAV8 (Figure 5B). Compared to AAV8, the AAV7 VP showed different surface loop conformations in VR-I (1 aa deletion), VR-IV (1 aa insertion), and VR-VII (1 aa deletion), respectively. In fact, these AAV7 loops are unique among all the available AAV serotype capsid structures. VR-I of AAV7 is structurally most similar to AAV1 and AAV6, without deletions or insertions but with amino acid variations resulting in Cα distance variation of up to 3 Å. AAV7’s VR-VII is the shortest loop among all AAV serotypes with a 1 aa deletion compared to AAV1-AAV4, and AAV6-AAV13 and a 4 aa deletion compared to AAV5. AAV7 vectors were shown to result in high transduction efficiencies of the CNS and spinal cord after delivery into the cerebrospinal fluid or intravenously [63,64]. This indicates that AAV7 might be able to cross the blood–brain barrier (BBB). However, the proposed residues in AAVrh.10 reported to be responsible for this phenotype [17] are only partially conserved in AAV7, e.g., S269, but not N472, where AAV7 has a threonine. More research is needed to determine if AAV7 has the ability to cross the BBB. AAV7 was shown to bind to several AAV8 antibodies, which are termed ADK8, HL2381, and HL2383 [65,66]. These antibodies utilize AAV8’s VR-VIII as its epitope [67,68]. The observed cross-reactivities can be explained by the high structural conservation of VR-VIII between AAV7 and AAV8 (Figure 6A).

For AAV11 and AAV12, the closest related AAV serotype is AAV4 (Figure 5A) based on VP1 or VP3 amino acid sequence ranging from 78 to 81% aa sequence identity (Figure 5B). However, the sequence identity of AAV11 and AAV12 to each other is slightly higher (84–87%). Surprisingly, the structural identity between AAV11 and AAV12 is 98%, which is only surpassed by AAV1 and AAV6 with 99% sequence and structural identity (Figure 5B). Compared to all other AAV serotypes, the VP3 sequence identity ranges from 51 to 59% (Figure 5B). Consequently, the AAV4 VP is structurally more similar to AAV11 and AAV12 when superposed (Figure 6B) with a structural identity of 91–92% compared to all other AAV serotypes with structural identities ranging from 63 to 79% (Figure 5B). In particular, AAV4, AAV11, and AAV12 share the insertion in VR-V and the alternative conformation of VR-IV (Figure 6B). The overall Cα RMSD of AAV11 and AAV12 to AAV4 is 0.68 and 0.70 Å with minor loop variations in VR-II, VR-III (1 aa deletion in AAV11 and AAV12), VR-VII, and VR-VIII (Figure 6B). When AAV11 and AAV12 are compared to each other, minor structural differences were observed in VR-II and VR-VII with an overall Cα RMSD of 0.56 Å. We propose that rather than clonal isolates, these three viruses, AAV4, AAV11, and AAV12 (Figure 5A), should be grouped into a new clade G.

While for AAV4, α2–3 linked sialic acid is described as a receptor [22], the receptor for AAV11 and AAV12 is unknown. For AAV12, HSPG and sialic acids were excluded as a receptor [62], and no binding to the available glycans on an array was shown [29]. Amino acids in AAV4 were suggested to be involved in sialic acid binding, which involves residues in VR-V, VR-VI, and VR-VIII [69]. The amino acids in VR-V and VR-VI are conserved structurally and in residue type in AAV11 and AAV12, unlike those in VR-VIII, which may be the reason why AAV11 and AAV12 do not bind sialic acids. Overall, AAV11 and AAV12 vectors have been rarely used for gene delivery purposes; however, AAV11 was described to possess a tropism for the spleen and smooth muscle [61,70], whereas AAV12 was shown to transduce nasal epithelia efficiently [71].

An interesting difference between AAV4, AAV11, and AAV12 for AAV vector production is the requirement for the assembly activating protein (AAP) for capsid assembly [72,73]. While AAV12 is dependent on the presence of AAP, AAV4 and AAV11 are not. An analysis of residues shared between AAV4 and AAV11 but not with AAV12 revealed a total of 23 aa. Previous studies suggested that interior residues are involved in the AAP function [74,75]. Only four of 23 aa differences are located in the interior of the capsid, A301S, A338T, I619V, and R688H (first aa type = AAV4/11, second aa type = AAV12). Of these aa positions, 619 is likely not the determining factor, since AAV11’s isoleucine is conserved in most AAV serotypes. A301S and R688H are located near the 2-fold symmetry axis, which is the previously suggested region for AAP binding [74]. More research is needed to confirm the importance of the residues for capsid assembly. 

For AAV13, the closest related AAV serotype is AAV3 (Figure 5A) based on VP1 or VP3 amino acid sequence ranging from 94 to 95% aa sequence identity, which is followed by AAV2 with 88–90% (Figure 5B). Nonetheless, when superposed, AAV13 is structurally slightly more similar to AAV2 compared to AAV3 (Cα RMSD: 0.65 Å vs. 0.72 Å and structural identity: 96% vs. 92%) (Figure 4B, Figure 5B, and Figure 6C). In addition to the significant difference in VR-IV caused by 2 aa deletion of AAV13 relative to AAV3, there are also minor variations in VR-I, VR-II, and VR-V (Figure 6C). Common to all of these three AAV serotypes is their ability to bind to HSPG [25,28,29,76] and to the A20 antibody [77]. For AAV13, a critical aa for this binding is K528, which is not present in AAV2 or AAV3 [28]. This residue is located on the side of the 3-fold protrusion within VR-VI. The mutation of K528 to glutamic acid results in the inability to bind HSPG [28,29]. Interestingly, this residue is in a structural equivalent position to AAV6-K531 reported to be important for HSPG binding of AAV6 [78]. AAV13 vectors have currently not been used for gene delivery purposes. Thus, more research is needed to determine its tropism and transduction efficiency.

## 4. Conclusions

This study determined the capsid structures of AAV7, AAV11, AAV12, and AAV13, thereby completing the panel of available structures for all currently defined AAV serotypes. While these capsids conserve the AAV capsid features such as the 5-fold channels, protrusions around the 3-fold symmetry axes, depressions at the 2-fold axes, as well as nucleotide binding pocket, they also display surface loops that are not found in any other AAV serotype structure. These separate AAV7 from its closest related AAV members contained within clade E (such as AAV8, AAV10, or AAVrh.10). 

AAV11 and AAV12 share structural similarity to AAV4 with loop conformations that are also not found in other AAV serotypes. Thus, while not defined as such, AAV4, AAV11, and AAV12 might form a separate clade. Lastly, AAV13 with AAV3 as its closest related AAV serotype shares structural similarity to both AAV2 and AAV3. It likely belongs to clade C, which was previously described to contain AAV2–AAV3 hybrid members.

The definition of clades suggests antigenic specificity, with members being cross-reactive [3]. However, recent data shows that members of different clades cross-react, and thus the clade definition requires revisiting [66]. The completion of the AAV serotype structural atlas, providing visualization of the conserved and variable regions, shows that the serotypes can also be grouped based on structural morphology. These structures provide a template for engineering the AAV capsids for targeted tissue tropism and the escape of recognition by host antibodies toward improved vector efficacy.

## Figures and Tables

**Table 1:** Summary of data collection, image processing, and refinement statistics.

Cryo-EM Data and Refinement Parameter	AAV7		AAV11		AAV12		AAV13	
	Full	Empty	Full	Empty	Full	Empty	Full	Empty
Total number of micrographs	271		1251		1629		1582	
Defocus range (µm)	0.8–2.0		0.8–3.0		1.0–3.0		0.8–3.0	
Total electron dose (e^−^/Å^2^)	60		60		60		60	
Frames/micrograph	71		45		50		50	
Pixel size (Å/pixel)	1.08		0.85		1.08		1.08	
Capsids used for final map	4695	40,988	10,429	118,351	40,764	220,137	6794	56,962
Resolution of final map (Å)	3.16	2.96	3.15	2.86	2.67	2.54	3.00	2.76
**Refinement Statistics**								
Map CC	0.871	0.899	0.859	0.863	0.864	0.868	0.856	0.864
RMSD bonds (Å)	0.01	0.01	0.01	0.01	0.01	0.01	0.01	0.01
RMSD angles (°)	0.79	0.83	0.82	0.99	0.91	0.94	0.88	0.95
All-atom clashscore	8.83	7.78	9.29	10.38	8.91	7.96	8.26	8.99
**Ramachandran plot (%)**								
Outliers	0	0	0	0	0	0	0	0
Allowed	2.3	1.7	2.3	1.7	1.5	1.5	1.9	2.1
Favored	97.7	98.3	97.7	98.3	98.5	98.5	98.1	97.9
Rotamer outliers	0	0	0	0	0	0	0.2	0
C_β_ deviations	0	0	0	0	0	0	0	0
